# Supplementary material for: Electrospun Composite Nanofibrous Materials Based on (Poly)-Phenol-Polysaccharide Formulations for Potential Wound Treatment
Source: Materials (Basel). 2020 Jun 9;13(11):2631. doi: 10.3390/ma13112631 (PMC7321623; doi:10.3390/ma13112631)
Supplement: Supplementary file 1 [file materials-13-02631-s001.pdf]

materials

Electrospun Composite Nanofibrous Materials Based on (Poly)-Phenol-Polysaccharide Formulations for Potential Wound Treatment

Lidija Fras Zemljič [1,*], Uroš Maver [2], Tjaša Kraševac Glaser [1], Urban Bren [3], Maša Knez Hrnčič [3], Gabrijela Petek [4] and Zdenka Peršin [1,5]

1 Laboratory for Characterization and Processing of Polymers, Faculty of Mechanical Engineering, University of Maribor, Smetanova 17, SI-2000 Maribor, Slovenia; tjasha.sternad@gmail.com (T.K.G.); zdenka.persin@gmail.com (Z.P.)
2 Faculty of Medicine, Institute of Biomedical Sciences and Department of Pharmacology, University of Maribor, Taborska ulica 8, SI-2000 Maribor, Slovenia; uros.maver@um.si
3 Faculty of Chemistry and Chemical Engineering, University of Maribor, Smetanova 17, SI-2000 Maribor, Slovenia; urban.bren@um.si (U.B.); masa.knez@um.si (M.K.)
4 Faculty of Electrical Engineering and Computer Science, University of Maribor, Smetanova 17, SI-2000 Maribor, Slovenia; gabrijela.petek@um.si
5 The BISTRA Scientific Research Centre Ptuj, Slovenski trg 6, SI-2250 Ptuj, Slovenia
* Correspondence: lidija.fras@um.si; Tel.: +386-2-220-7909

Received: 23 April 2020; Accepted: 5 June 2020; Published: date

Weibull model and Korsmeyer-Peppas model data

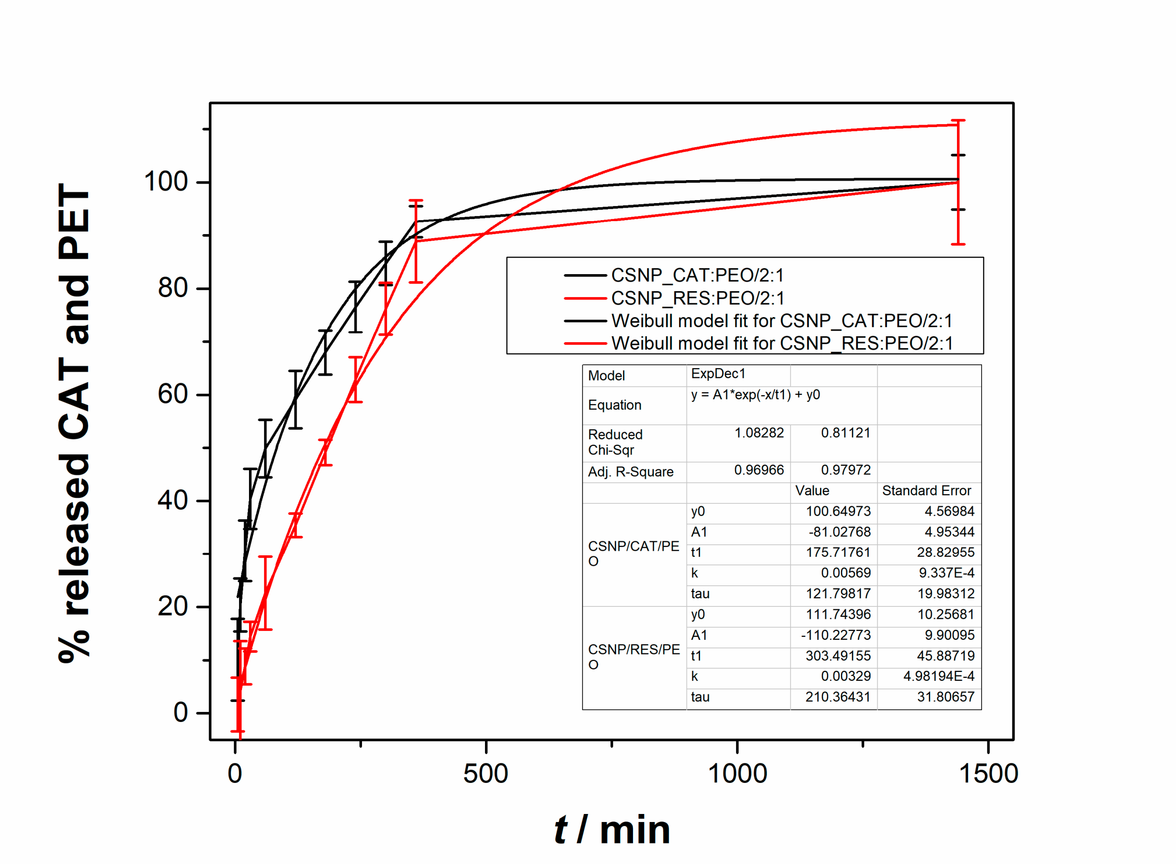

Model	ExpDec1		
Equation	y = A1*exp(-x/t1) + y0		
Reduced Chi-Sqr	1.08282	0.81121	
Adj. R-Square	0.96966	0.97972	
		Value	Standard Error
CSNP/CAT/PEO	y0	100.64973	4.56984
	A1	-81.02768	4.95344
	t1	175.71761	28.82955
	k	0.00569	9.337E-4
	tau	121.79817	19.98312
CSNP/RES/PEO	y0	111.74396	10.25681
	A1	-110.22773	9.90095
	t1	303.49155	45.88719
	k	0.00329	4.98194E-4
	tau	210.36431	31.80657

Figure S1. Time dependent change in the percentage of the released incorporated drug with Weibull model fitting graph.

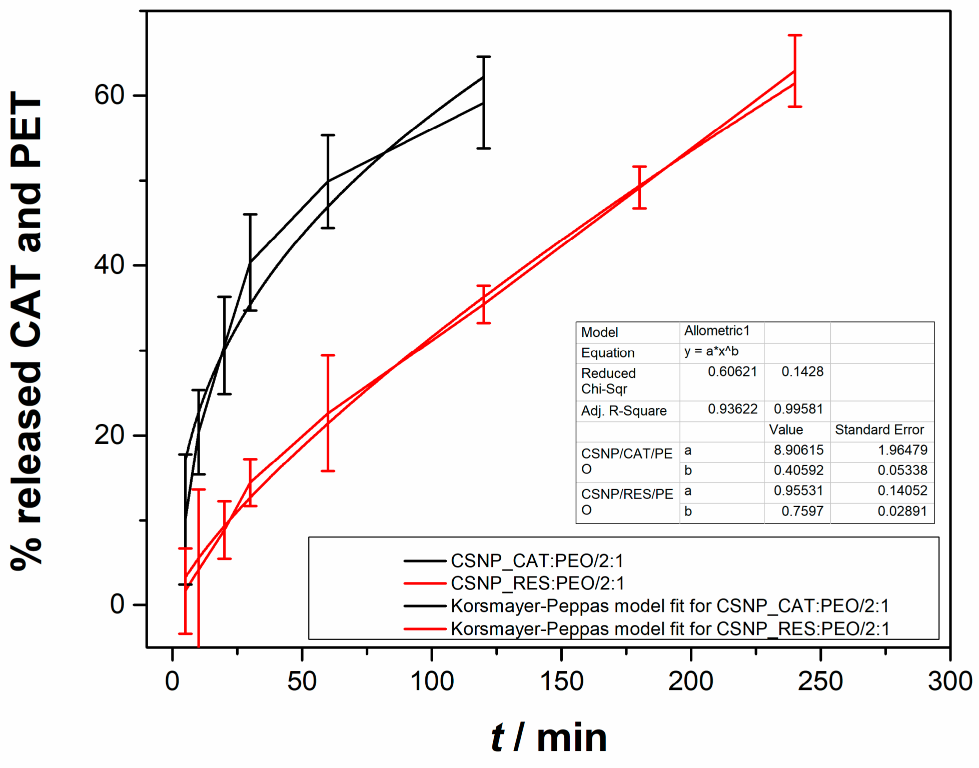

Figure S2. Time dependent change in the percentage of the released incorporated drug with Korsmeyer-Peppas model fitting graph.
